# Traumatic Bilateral Bronchial Injury Requiring Pneumonectomy

**DOI:** 10.7759/cureus.76145

**Published:** 2024-12-21

**Authors:** Kevin J Hsu, Kiyoshi Chandler, Charles Fasanya, John W Hsu

**Affiliations:** 1 Trauma and Acute Care Surgery, Good Samaritan University Hospital, West Islip, USA

**Keywords:** bilateral bronchial injury, blunt thoracic trauma, endobronchial stenting, traumatic pneumonectomy, venovenous extracorporeal membrane oxygenation

## Abstract

High-energy blunt thoracic trauma is a highly morbid condition. When a pneumonectomy is required in such a setting, the mortality rate increases significantly. Here, we present a case of a motor vehicular crash (MVC) in which the patient suffered bilateral bronchial injuries requiring emergent thoracotomy, pneumonectomy, bronchial stenting, and initiation of venovenous extracorporeal membrane oxygenation (VV ECMO). The patient ultimately survived his injuries and was able to fully recover. We review the relevant literature in regard to the use of pulmonary stenting and VV ECMO in the setting of thoracic trauma management.

## Introduction

A severely morbid event is that of blunt force trauma resulting in a tracheobronchial disruption and subsequent pneumonectomy. Recent reviews of the literature suggest that mortality rates in this situation range from 50% to 100% [1]. Although uncommon, with incidences of a traumatic pneumonectomy at 0.01% [2], this type of traumatic injury is morbid due to both the extent of the injury and the development of a post-pneumonectomy physiology [3]. Any blunt thoracic trauma requiring lung resection is associated with complications such as right heart failure, air embolism, pulmonary infections, acute respiratory distress syndrome (ARDS), refractory hypoxia, and death [4]. Even rarer is a bilateral injury to the tracheobronchial tree. Historically, most patients with tracheobronchial injuries do not survive the trip to the trauma center [5]. Despite these dismal outcomes, we report on a 30-year-old man presenting to our institution who was involved in a high-speed MVC - resulting in a bilateral tracheobronchial injury requiring a pneumonectomy on one side, bronchial stenting on the other, and initiation of ECMO - and survived. In this severely injured cohort, we postulate the need to treat with an aggressive, early, multidisciplinary approach to maximize chances of survival.

## Case presentation

**Figure 1:**
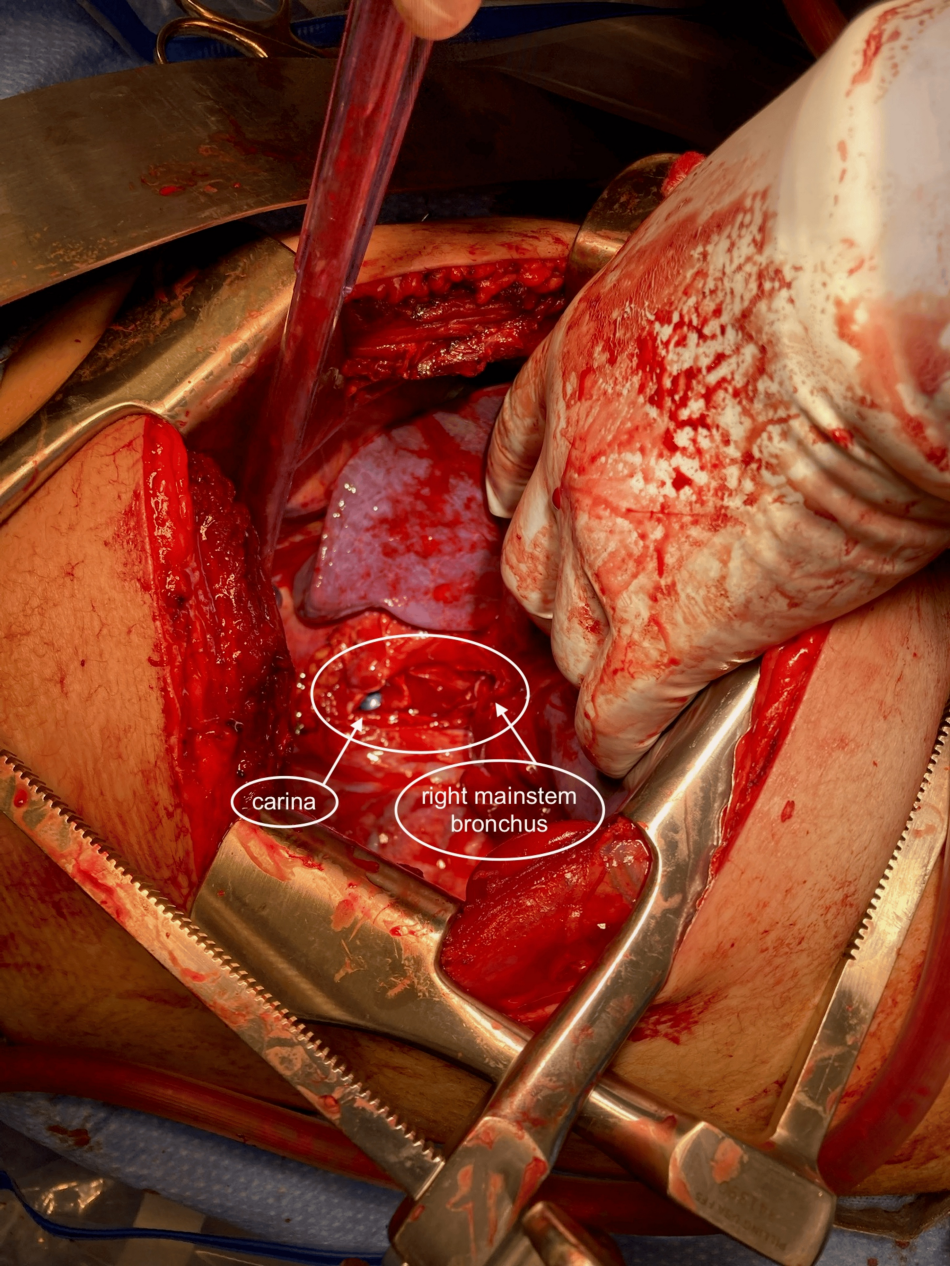
An intraoperative photo showing the avulsed right bronchus at its root. Note the endotracheal tube at the level of the carina. Due to patient instability and wide defect, a pneumonectomy was performed and the bronchus was oversewn.

Our patient is a 30-year-old man who was involved in a high-speed MVC in which he rear-ended a parked flatbed truck on the side of the highway. He suffered severe deceleration injuries to his upper torso and extremities. He presents obtunded, intubated in the field, with a reported initial Glasgow Coma Scale (GCS) of 6. Advanced trauma life support (ATLS) protocol was initiated and the patient was found to have a massive air leak and taken emergently to the OR for a thoracotomy and repair of an anticipated bronchial disruption. On the way to the OR, the secondary survey was completed and the patient was also found to have a basilar skull fracture, open comminuted distal humerus fracture, sternal fracture, bilateral first rib fractures, right-sided multilevel rib fractures, and pulmonary contusion of his left lung. In the OR, an avulsed right bronchial root was found and attempts at a primary repair were abandoned due to patient instability. A traumatic pneumonectomy was performed and the root of the bronchus was oversewn. The avulsed right bronchus is shown in Figure 1.

The patient’s open fractures were washed out during the same procedure and his other injuries were addressed subsequent to his initial surgery by the appropriate surgical services.

The patient was admitted to the surgical critical care service where ventilator support was initiated. The pulmonary insult was significant and by the end of the first week, the patient developed ARDS and was using maximum mechanical ventilator support. Due to persistent hypoxia and hypercapnia, extracorporeal membrane oxygenation (ECMO) was initiated. A bedside tracheostomy was performed, and on bronchoscopy, a concomitant left bronchial injury was found. At this point the patient’s prognosis was poor, and given a persistent air leak in a left-sided chest tube, a self-expanding covered metal stent was placed across the left bronchial tear.

The patient continued to be in critical condition with minimal progression, so arrangements were made to transfer to a quaternary care center with the consideration of a possible lung transplant should the patient’s ventilatory status deteriorate further. Upon transfer, his condition improved, with subsequent removal of the bronchial stent and decannulation from ECMO. Ultimately, the patient was able to be weaned from the vent and then from his tracheostomy. At this point, he was ambulatory and discharged to a rehabilitation facility where he made a full recovery and was subsequently discharged home.

## Discussion

This case demonstrates the presentation of severe blunt thoracic trauma requiring emergent thoracotomy and subsequent traumatic pneumonectomy. As has been described in the literature [3], extra-pulmonary injuries usually accompany severe lung and bronchial tree injuries, causing further complications. In this case, the patient suffered bilateral bronchial injuries with concomitant high-impact thoracic cage injuries, long bone injuries, and a basilar skull fracture. There were no intra-abdominal solid organ injuries.

Reports on traumatic pneumonectomy cite a mortality rate of 50-100% [1]. Most centers acknowledge the difficulties and risks of delay in diagnosis [6-8]. In this case, we present both instances of a massive air leak on the initial workup which led to an aggressive, early surgical intervention in a trauma code. In addition, a more subtle development of persistent air leaks after the initial pneumonectomy and subsequent contralateral thoracostomy tube placement led to the bronchopleural fistula diagnosis.

We report the use of pulmonary stenting as an adjunct for acute and subacute bronchopleural fistulas related to thoracic trauma. Given the severity of the injury, deployment of a pulmonary stent may allow the patient to avoid a thoracotomy and possibly a second surgery, as in this case. Further, a review of the literature demonstrates a lack of publication on the use of pulmonary stenting in acute thoracic trauma. Currently, stents are mainly used for complications after oncologic surgery and iatrogenic tracheal membrane injuries [9,10]. The criteria for stenting include a stable patient with impending respiratory failure. In this case, the patient was stable after the pneumonectomy with a persistent air leak on the contralateral side. Our decision to proceed with pulmonary stenting was driven by expertise in pulmonary stenting at our institution and a frail, but stable, young patient.

The overall morbidity that our patient suffered stems from blunt forces causing contusion to the remaining lung and concomitant injuries. Specifically, this patient had injuries to the contralateral lung and bronchus, leading to the development of severe ARDS with persistent hypoxia and hypercapnia despite maximal ventilator support. The patient proceeded to be placed on ECMO by the cardiac surgery service. Recently, there have been reports of utility and success in the management of severely traumatized patients with the use of venovenous (VV) ECMO with a recognition of a need to designate traumatic patients being treated with this modality [11]. The early experience with ECMO and trauma have led to discussions on problems such as the role of VV ECMO versus venoarterial (VA) ECMO, time to institute ECMO, and anticoagulation strategies while on the circuit [12]. However, with ongoing reassessment of the indications and contraindications of ECMO treatment, a clinical consensus remains of the lifesaving potential of ECMO application in the severely injured trauma patient [13,14].

## Conclusions

The successful treatment of this case, involving bilateral severe blunt thoracic injury resulting in a pneumonectomy, required a multidisciplinary, multi-center collaboration. This patient was saved by an aggressive deployment of a myriad of service lines across two hospital systems including pulmonary stenting and VV ECMO. This shows that even a uniformly fatal injury can be salvaged with an aggressive, coordinated, “all hands” approach to care.
